# Metabolic Profiling and Potential Taste Biomarkers of Two Rambutans during Maturation

**DOI:** 10.3390/molecules28031390

**Published:** 2023-02-01

**Authors:** Hao Deng, Guang Wu, Li Guo, Fuchu Hu, Liying Zhou, Bin Xu, Qingchun Yin, Zhe Chen

**Affiliations:** 1 Key Laboratory of Tropical Fruit and Vegetable Cold-Chain of Hainan Province, Institute of Agro-Products of Processing and Design, Hainan Academy of Agricultural Sciences, Haikou 571100, China; 2 Sanya Institute, Hainan Academy of Agricultural Sciences, Sanya 572025, China; 3 Key Laboratory of Genetic Resources Evaluation and Utilization of Tropical Fruits and Vegetables (Co-construction by Ministry of Province), Ministry of Agriculture and Rural Affairs, Haikou 571100, China; 4 Key Laboratory of Tropical Fruit Tree Biology of Hainan Province, Institute of Tropical Fruit Trees, Hainan Academy of Agricultural Sciences, Haikou 571100, China; 5 Key Laboratory of Tropical Fruits and Vegetables Quality and Safety for State Market Regulation, Hainan Institute for Food Control, Haikou 570314, China

**Keywords:** rambutan, sugar, acid, biomarker, widely targeted metabolomics, maturation

## Abstract

The metabolite-caused taste variation during rambutan maturation is unknown due to a lack of systematic investigation of all components. In this study, three growing stages, including unripe (S1), half-ripe (S2), and full-ripe (S3) BY2 and BY7 rambutans were compared and profiled by UPLC–MS/MS-based widely targeted metabolomics analysis. We demonstrated that the sugar-acid ratios of two rambutans were greatly improved between the S2 and S3 stages. A total of 821 metabolites were identified, including 232, 205, 204, and 12 differential metabolites (DMs) in BY2-S1 vs. BY2-S2, BY2-S2 vs. BY2-S3, BY7-S1 vs. BY7-S2, and BY7-S2 vs. BY7-S3, respectively. A correlation analysis showed that gamma-aminobutyric acid (GABA) could be the sugar-acid ratio biomarker of BY2 rambutan. Methionine (Met), alanine (Ala), and S-methyl-L-cysteine (SMC) could be total amino acid biomarkers of BY2 and BY7 rambutans. In addition, UPLC-MS/MS-based quantitative verification of the above biomarkers exhibited the same variations as metabolomics analysis. This study not only provides useful nutritive information on rambutans but also valuable metabolic data for rambutan breeding strategies.

## 1. Introduction

Rambutan (*Nephelium lappaceum* L.) is a popular tropical fruit from the family Sapindaceae [1]. The pulp has a similar flavor to lychee (Litchi, chinensis), but is less aromatic, and the skin is covered in unique hair-like spinterns [2]. The color of this mature fruit varies from yellow to red, depending on the cultivar. Rambutan is an important commercial fruit in Asia and has gained great popularity among worldwide customers due to its splendid sweet-sour flavor and exotic appearance. The continuing spread of rambutan to markets has led to an increased research effort into new cultivar breeding and cultivation. The commercial maturity of this fruit generally depends on skin color as well as eating quality. Rambutan is a nonclimacteric fruit and will not continue to ripen after being collected from trees [2]. Consequently, rambutan must be harvested when the taste-related qualities and appearance have reached an optimal balance. If the fruit is harvested too early, the pulp is acidic, while the pulp of overripe fruit is drier, firmer and bland. 

Many studies have investigated the crucial eating qualities or nutrients of different rambutan cultivars but have mainly focused on specific classes. It has been reported that rambutan contains high levels of sugars, mainly including sucrose (5.38–10.01%), fructose (1.75–3.18%) and glucose (1.72–2.43%). Citric acid is the predominant organic acid in both fruit and underutilized wild rambutan cultivars [3]. Other organic acids, including tartaric, malic, succinic, and lactic acids, are also reported in rambutan [4]. Alanine (Ala) is a major amino acid found in rambutan, and gamma-aminobutyric acid (GABA) levels increased as the fruit matured [5]. However, limited information about the comprehensive comparison of sugars, organic acids, amino acids, and related metabolites during maturation is available. 

Metabolomic profiling is a crucial strategy to analyze nutrient components at the molecular level [6]. Investigating metabolite-caused variations between cultivars is also important for rambutan breeding with superior edible and nutritional quality. Currently, an ultra-performance liquid chromatography-tandem mass spectrometry (UPLC–MS/MS)-based widely targeted metabolomics analysis has been successfully used for the simultaneous detection of a vast number of metabolites, and rapidly provides valuable molecular information of components in different cultivars [7]. With the aid of chemometric statistical analysis, widely targeted metabolomics was also used for the further objective and reliable detection of molecular biomarkers [8]. Due to the absence of the comprehensive metabolic profiling of rambutan, little evidence has been found associating sugar, acid, and amino acid biomarkers in different cultivars during maturation.

In this study, we obtained a special rambutan cultivar, named Baoyan 2 (BY2), with a unique yellow pericarp and excellent flavor depending on the maturation stage. Compared with the widely cultivated Baoyan 7 (BY7) rambutan, the main taste qualities, including sugars, acids, and amino acids during BY2 maturation, were investigated. A UPLC-MS/MS-based widely targeted metabolomics analysis was then performed to identify and quantify all metabolites, especially sugar, acid, and amino acid related metabolites. With the aid of multivariate statistical and correlation analysis, the characteristic metabolites and potential taste biomarkers were screened and verified. This study not only provides useful nutritive information on rambutans, but also valuable metabolic data for rambutan breeding strategies.

## 2. Results and Discussion

### 2.1. Basic Qualities of BY2 and BY7 Rambutans at Different Growth Stages

The color contribution index (CCI) is a comprehensive color indicator used to measure fruit pericarp. A positive CCI value indicates a yellow-red color, while a negative CCI value represents a blue-green color [9]. As shown in Table S1, the CCI values of BY2 at the S2 and S3 growing stages were −1.7 and 0.3, respectively, with the pericarp color turning from green to yellow. The pericarp of BY7 rambutan became strongly red from yellow, with CCI values increasing from 8.4 to 30.2. 

The total soluble solid (TSS), pH, soluble sugar, titratable acid, and sugar-acid ratio are basic qualities of fruit and are widely chosen for evaluation of maturation degree for harvest [10]. As shown in Table S1, TSS and pH gradually increased during the unripe (S1), half-ripe (S2), and full-ripe (S3) stages of the two rambutans. The sugar-acid ratios of BY2 and BY7 significantly (*p* < 0.05) increased from 28.6 and 15.5 at the S2 stage, to 94.5 and 74.0 at S3 stage, respectively. The sharp decline of titratable acid from 0.52% to 0.17% was the main reason for the sugar-acid ratio variation of BY2 between the S2 and S3 stages. In contrast, the soluble sugar content of BY7 rambutan was significantly (*p* < 0.05) increased during the same growing stages. The above findings demonstrated that the sweet taste of both cultivars was greatly improved between the S2 and S3 stages. However, the degradation of acids in BY2 and the accumulation of sugars in BY7 during these growing stages were obviously different.

### 2.2. Sugars and Organic Acids of BY2 and BY7 Rambutans at Different Growth Stages

To investigate the reason for sugar-acid ratio changes in the two cultivars during maturation, three sugars and seven organic acids were compared. As shown in Table S2, sucrose was the predominant sugar in BY2 rambutan, the contents of which were 70.8, 78.5, and 83.9 g/kg at the three growing stages, respectively. Similarly, the contents of sucrose in BY7 rambutan increased gradually during ripening. Sucrose and fructose in BY7 rambutan significantly (*p* < 0.05) increased from 82.4 and 16.0 g/kg at S2 to 97.2 and 18.5 g/kg at S3, respectively. However, the two main sugars of BY2 remained relatively stable. Sucrose, fructose, and glucose are the main sugars in fruit and vary by maturation and cultivar [11,12]. A previous study reported that rambutan contains high levels of sugars, mainly sucrose (5.38–10.01%), fructose (1.75–3.18%) and glucose (1.72–2.43%) [3]. Our findings are consistent with that study.

Three predominant organic acids of BY2 rambutan, including lactic, citric, and malic acids, decreased significantly (*p* < 0.05) from 14.2, 5.9, and 7.4 g/kg at the S2 stage to 10.2, 2.0, and 5.7 g/kg at the S3 stage, respectively. These changes are consistent with the sharp decline in titratable acid in BY2 rambutan in Table S1. In contrast, lactic and malic acids in BY7 rambutan increased during the same growth periods. Recently, citric, tartaric, malic, succinic, and lactic acids have been reported in rambutans in previous studies [4]. Interestingly, two unique organic acids, adipic and fumaric acids, were first detected and analyzed by us in rambutan. The above findings proved that the sharp decline in lactic, citric, and malic acids contributed primarily to the sugar-acid ratio increase and taste improvement during BY2 rambutan maturation. Meanwhile, sucrose and fructose accumulation in BY7 rambutan played an important role in sweet taste enhancement from the half-ripe to full-ripe growth stage. 

### 2.3. Amino Acids of BY2 and BY7 Rambutans at Different Growth Stages

Amino acids, the main primary and secondary metabolites in fruit, are beneficial to human health and determine crucial fruit quality [13]. Furthermore, amino acids may be an important contributory factor to fruit taste. For instance, arginine (Arg) and asparagine increase sweetness, while aspartate (Asp) enhances sourness [14]. As shown in Table S3, nineteen amino acids, including seven essential amino acids, showed apparent changes during fruit maturation. The total amino acid contents of the two cultivars increased significantly to reach the maxima of 1438.7 and 1207.2 mg/kg at the S2 stage, respectively. The amino acids then declined slightly from the S2 to S3 stages. The majority of amino acids, including 16 amino acids in BY2 rambutan and 13 amino acids in BY7 rambutan, were consistent with the variation in total amino acid content during maturation. Interestingly, alanine (Ala) was the main amino acid in both BY2 and BY7 rambutan, and increased by 402.6% and 754.5% from unripe to half-ripe in the two cultivars. Ala is not only an important proteinogenic amino acid, but also a potential exogenous precursor of aromatic volatiles, and the most aromatic fruit cultivar was also found to have the highest Ala concentration at maturity [15]. 

In summary, the sugar-acid ratios and taste of BY2 and BY7 rambutans were greatly improved from the half-ripe to the full-ripe stages. The taste enhancement of BY2 rambutan mainly relied on the reduction of lactic, citric and malic acids. However, the increase in sucrose and fructose in BY7 rambutan primarily determined the sweet taste improvement at the same growth stages. In addition, the total amino acid contents of the two cultivars increased significantly to reach maxima of 1438.7 and 1207.2 mg/kg at the S2 stage, respectively. The majority of amino acids observed the same variation. To date, far too little attention has been given to the comparative analysis of all taste-related metabolites, especially sugars, organic acids, and amino acids at the different growth stages of rambutan. Therefore, a UPLC-MS/MS-based widely targeted metabolomics analysis was performed to reveal the taste component variations during maturation.

### 2.4. Widely Targeted Metabolomics Analysis

**Figure 1:**
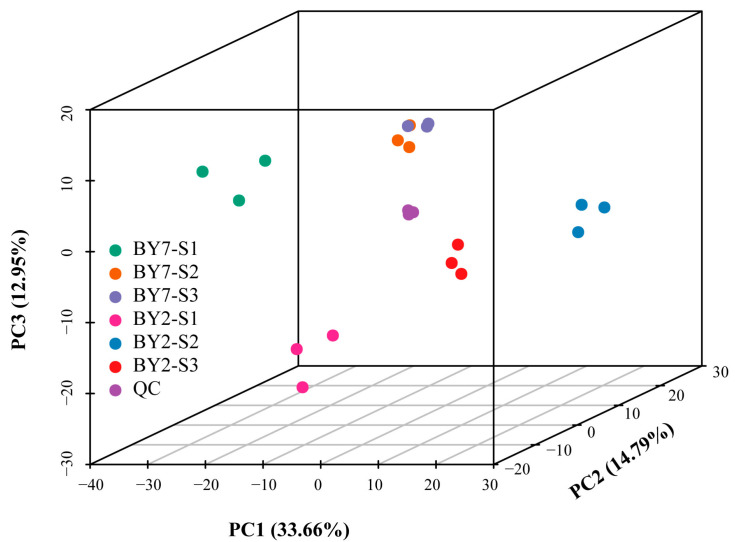
Principal component analysis of metabolites identified from BY2 and BY7 rambutans. Note: Each growth stage had three individual samples. Equal volumes of flesh samples were mixed for use as quality control (QC).

In total, 821 metabolites were identified, including sugars, organic acids, amino acids, flavones, and others. Principal component analysis (PCA), a multivariate statistical method for transforming the original correlated data into a new set of unrelated variables named principal components (PCs), is widely used for data exploration, pattern recognition, and data visualization [16]. As shown in Figure 1, the contributions of PC1, PC2, and PC3 were 34.66%, 14.79%, and 12.95%, respectively. The total contribution of these three PCs was 62.4%, which contained the majority of information for all metabolites. Six groups, including BY2-S1, BY2-S2, BY2-S3, BY7-S1, BY7-S2, and BY7-S3, were clearly separated, and the three samples within each group were closely clustered. Six samples of BY7-S2 and BY7-S3 were closed in PCA, indicating that the development of this cultivar was relatively slow during the half-ripe to the full-ripe stage. Equal volumes of all samples were mixed for use as quality control (QC), three samples of which were clustered in the center of all groups. The above findings proved that three replicates had good reproducibility, and all different growth stage samples of BY2 and BY7 had individual metabolite profiles. 

**Figure 2:**
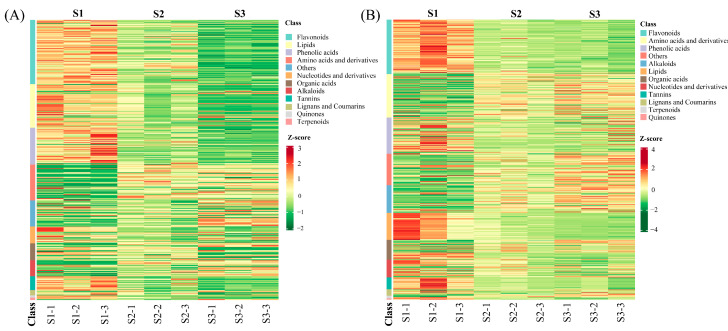
Hierarchical cluster analysis of metabolites identified from two rambutans. (**A**) HCA of metabolites identified from BY2 rambutan at three growth stages. (**B**) HCA of metabolites identified from BY7 rambutan at three growth stages. Note: The color from green (low) to red (high) indicates the level of each metabolite. The Z-score represents the deviation from the mean by standard deviation units.

A hierarchical cluster analysis (HCA) was applied to investigate the chemical class difference of all metabolites during maturation. As shown in Figure 2, all 821 metabolites were divided into 12 classes in both BY2 and BY7 rambutan, but the majority of them were flavonoids, lipids, phenolic acids, and amino acids. BY2 had more flavonoids and lipids, while amino acids and derivatives were more abundant in BY7 rambutan. Moreover, flavonoids, lipids, and phenolic acids were observed to gradually decrease from S1 to S3 in the two cultivars. In contrast, most amino acids were found to increase during ripening. The above PCA and HCA results both proved that the three growth stages of the two rambutans were three distinct groups with individual metabolite profiles. 

### 2.5. Identification and Classification of DMs

**Figure 3:**
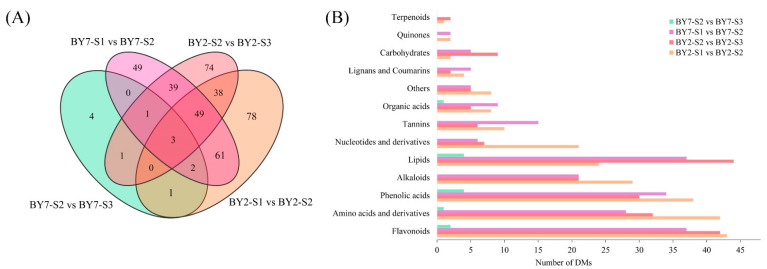
Venn diagram (**A**) and classification (**B**) of differential metabolites in two rambutans at three growth stages.

To identify differential metabolites (DMs) among the three growing stages, we selected metabolites based on VIP ≥ 1 and |Log_2_FC| ≥ 1.0. As shown in Figure 3A, there were 232, 205, 204, and 12 DMs in BY2-S1 vs. BY2-S2, BY2-S2 vs. BY2-S3, BY7-S1 vs. BY7-S2, and BY7-S2 vs. BY7-S3, respectively. The majority of DMs included flavonoids, amino acids and derivatives, phenolic acids, alkaloids, and lipids (Figure 3B). There were 4 and 74 unique DMs only found in BY7-S2 vs. BY7-S3 and BY2-S2 vs. BY2-S3, respectively. This proved that the nutritional and taste compounds of BY2 actively biosynthesized or decomposed from the S2 to S3 stages compared with BY7 rambutan. The least DMs were found in BY7 rambutan during the growing stage from the S2 to S3 stages, indicating that the two stages had a close relationship and that the improvement of this fruit quality was relatively slow. This result was consistent with findings of the PCA analysis (Figure 1A). Specifically, three DMs, including 3-O-p-coumaroylshikimic acid, L-homocysteine, and lysoPC18:1 (2n isomer), were found in two cultivars from the unripe to full-ripe stages; therefore, they could be classified as common DMs.

Phenolic compounds are not only one of the sour taste contributors, but they also play a vital role in defence responses, such as with their anti-ageing, anti-oxidant, and anti-bacterial activities in the human body. 3-O-p-coumaroylshikimic acid is a rare phenolic compound and is sporadically found in Phlomis cashmeriana [17] and Phegopteris connectilis [18] as a unique chemical constituent of plants. Here, 3-O-p-coumaroylshikimic acid was first reported in rambutan. It is a derivative of shikimic acid (shikimate), which is the essential precursor for three proteinogenic aromatic amino acids, including Tyr, Try, and Phe [19]. Furthermore, phenolic compounds, including simple phenols, phenolic acids, hydroxycinnamic acids, phenylacetic acid, flavonoids, lignins, and catechol melanins are either derived from these three amino acids or directly from shikimate pathway intermediates [20]. The present study proved that quinate dehydrogenase (QDH) and shikimate dehydrogenase (SDH) control the accumulation of quinic acid in fruit. SDH activity was highest in the early stages of fruit development compared with the late growing stage [21]. We speculated that highly active SDH played a crucial role in the decline in shikimic acid and 3-O-p-coumaroylshikimic acid, as well as the increase in organic acids in the unripe rambutan fruit. In contrast, the activity of SDH was relatively slow in half-ripe and full-ripe rambutan, resulting in a gradually increasing sugar-acid ratio of fruit. 

L-homocysteine is a sulfur-containing amino acid that is a crucial intermediate compound that actively participates in the biosynthesis of other amino acids, including Met, Ser, and Cys [22]. L-homocysteine showed a sharp decline from the S2 to S3 stage of BY2 rambutan by widely targeted metabolomics analysis. Meanwhile, Met and Ser were also decreased by 46.7% and 25.2%, respectively (Table S3). We speculated that the biosynthesis of amino acids in BY7 rambutan mainly occurred at the S1 and S2 stages, and the synthesis and decomposition of amino acids at the S3 stage were dynamically balanced. Thus, the total amino acids of BY7 rambutan at the S3 stage remained unchanged. In addition, L-homocysteine was consumed for the biosynthesis of amino acids at the early stage and declined to the minimum at the S3 stage. Therefore, the synthesis of amino acids, especially Ser and Met, was relatively stable at the S3 stage of BY7 rambutan.

### 2.6. Correlation Analysis of Taste Indicators and Metabolites

**Figure 4:**
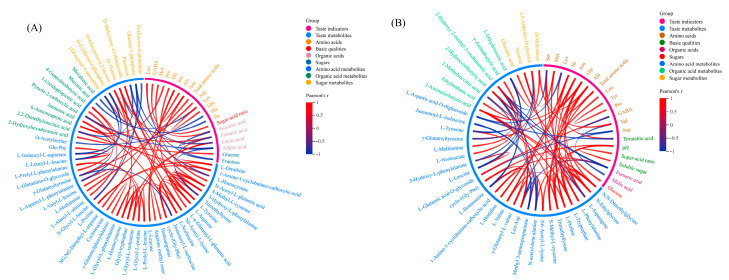
Correlation between taste indicators and metabolites in BY2 (**A**) and BY7 (**B**) rambutans. Note: Correlation coefficients were calculated by Pearson’s test (Pearson’s r > 0.9, *p* < 0.01).

To eliminate the effects of quantity on pattern recognition, we applied a log_10_ transformation of peak areas for all taste DMs, followed by a correlational analysis between them and taste indicators. As shown in Figure 4A, 21 crucial taste-related indicators, including 1 basic quality, 2 sugars, 4 organic acids, and 14 amino acids had significant (*p* < 0.01) correlations with 56 corresponding taste metabolites during BY2 rambutan maturation. The sugar-acid ratio, as the only basic quality indicator, showed a positive correlation (Pearson’s r = 0.9999) with gamma-aminobutyric acid (GABA). Glucose, the predominant sugar in BY2 rambutan, showed a positive correlation (Pearson’s r = 0.9999) with 2-hydroxyhexadecanoic acid. Leu had a positive correlation (Pearson’s r = 1) with succinic acid and a negative (Pearson’s r = −1) correlation with fructose. Total amino acids showed a positive correlation (Pearson’s r = 0.9999) with L-methionine in BY2 rambutan.

In total, 21 crucial taste-related indicators, including 4 basic qualities, 1 sugar, 2 organic acids, and 14 amino acids, had significant (*p* < 0.01) correlations with 38 corresponding taste metabolites during BY7 rambutan maturation (Figure 4B). Glucose, the only sugar indicator in BY7 rambutan, showed a positive correlation (Pearson’s r = 0.9999) with gluconic acid. Titratable acid had a positive correlation (Pearson’s r = 0.9999) with Lys and a negative (Pearson’s r = −0.9999) correlation with L-aspartic acid-O-diglucoside. The sugar-acid ratio showed a positive correlation (Pearson’s r = 0.9999) with soluble sugar. Total amino acids had positive correlations (Pearson’s r = 0.9999) with Ala and S-methyl-L-cysteine (SMC), respectively. Therefore, we speculated that GABA and Met could be the potential sugar-acid ratio and amino acid biomarkers of BY2 rambutan, respectively. Ala and SMC could be potential amino acid biomarkers of BY7 rambutan.

### 2.7. Verification of Potential Sugar-Acid Ratio Biomarker

**Figure 5:**
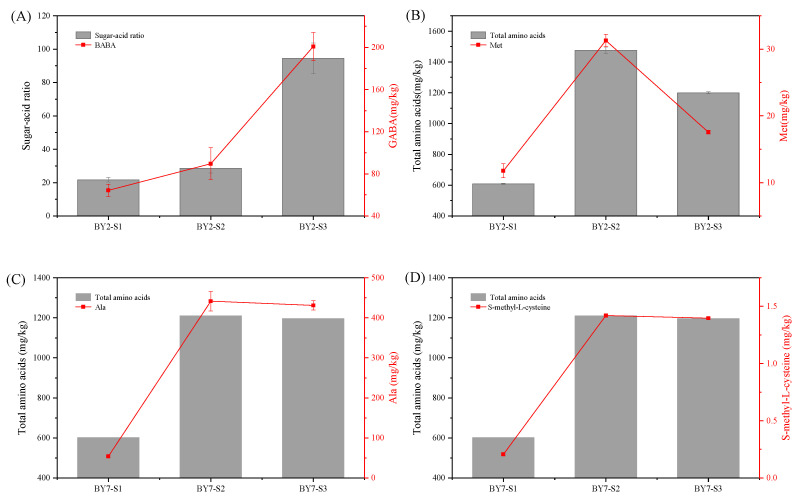
Quantitative verification of key taste makers of sugar-acid ratio (**A**) and total amino acids (**B**) in BY2 rambutan, and total amino acids (**C**,**D**) in BY7 rambutan.

Since widely targeted metabolomics can only relatively quantify the same metabolite of rambutan with different maturities, we conducted a UPLC-MS/MS based quantitative verification of the above potential taste biomarker again. As shown in Figure 5A, GABA of BY2 rambutan accumulated from 64.3 to 200.7 mg/kg during maturation, with the sugar-acid ratio increasing concomitantly from 21.7 to 94.5. This result was consistent with the correlation analysis. Thus, GABA could be a sugar-acid ratio biomarker of BY2 rambutan. 

Generally, an appropriate sugar-acid ratio is vital to fruit flavor and taste, and usually improves gradually during maturation [23]. Sugars are not only energy for metabolism, but also signaling material of plant development [24]. A prior study revealed a strong accumulation of GABA and a 10-fold increase in glutamate decarboxylase activity during the early growing stage of cherry tomato after anthesis. GABA plays an important role in pH regulation by acting as a sink for excess protons in the cytoplasm. The continuous production of GABA during fruit maturation may reflect the continuous need to counteract the pH-perturbing effect of organic acid accumulation [25]. Therefore, the pH of BY2 rambutan was observed to significantly (*p* < 0.05) increase with the accumulation of GABA during maturation.

### 2.8. Verification of Potential Amino Acid Biomarkers

As shown in Figure 5B,D, Met and SMC changed similarly to total amino acids in BY2 and BY7 rambutan, respectively. SMC, a natural cysteine-containing compound found in garlic and onion, has anti-inflammatory effects for alcoholic liver disorders [26], anti-obesity [27], and anti-lung cancer [28]. It was first reported in rambutan by us. Met and SMC are sulfur-containing amino acids and their derivates, which are important intermediate compounds that actively participate in the biosynthesis of other amino acids, including Ser, Cys, and homocysteine [22]. Met showed a 43.9% sharp decline in the half-ripe to full-ripe stage of BY2 rambutan by UPLC-MS/MS analysis. Ser was also found to decrease by 25.2% in the same growing stages (Table S3). As shown in Figure 5C, Ala increased significantly with total amino acids in early growing stages and was retained stably during the half-ripe to full-ripe stages in BY7 rambutan. Therefore, Met could be a biomarker of the total amino acids of BY2 rambutan, and Ala and SMC could be BY7 rambutan biomarkers.

Fruits are important sources of amino acids, which are the building blocks of proteins in the human body. Some essential amino acids cannot be biosynthesized by humans, so they must be obtained from foods [16]. In total, we found that two rambutans contained high amounts of 19 amino acids, including seven amino acids (Met, Lys, Ile, Leu, Phe, Thr, and Val) that are essential for humans. Although the total amino acids accumulated similarly in the two rambutans during the early growth stages, their biosynthesis, especially the biosynthesis of taste amino acids, i.e., Gly and Ala, were obviously different during maturation. Ala and Gly have strong sweetness because they both have the proton donors (AH) and proton acceptors (B) in molecules. The distance between AH and B is 2.5–4.0 Å. It was proven that the sweet sensation occurs when the AH and B of these amino acids form hydrogen bonds to the B and AH sites of their receptors in the tongue [29]. Val, Leu, Trp, and Phe generate strong and sweet tastes similarly. Therefore, the difference in these taste amino acids played an important role in the formation of the sweet taste of rambutan during ripening. Due to the sample constrains, this paper cannot provide a comprehensive review of all rambutans cultivated in China. The molecular mechanism of those biomarkers in regulating sugars and acids accumulation during rambutan maturation need to be further investigated.

## 3. Materials and Methods

### 3.1. Chemicals and Reagents

Chromatographic grade fructose, glucose, and sucrose were purchased from the National Institute of Metrology (Beijing, China). Chromatographic grade malic acid, lactic acid, tartaric acid, citric acid, succinic acid, and fumaric acid were purchased from Achemtek Co., Ltd. (Worcester, MA, USA). Chromatographic grade adipic acid was purchased from Alta Scientific Co., Ltd. (Tianjin, China). Chromatographic grade 21 amino acid mix solutions, including Ala, Met, and GABA were purchased from Achemtek Co., Ltd. (Worcester, MA, USA). Chromatographic grade S-methyl-L-cysteine was purchased from Shanghai Yuanye Bio-Technology Co., Ltd. (Shanghai, China). All others were of analytical reagent grade.

### 3.2. Materials and Sampling

**Figure 6:**
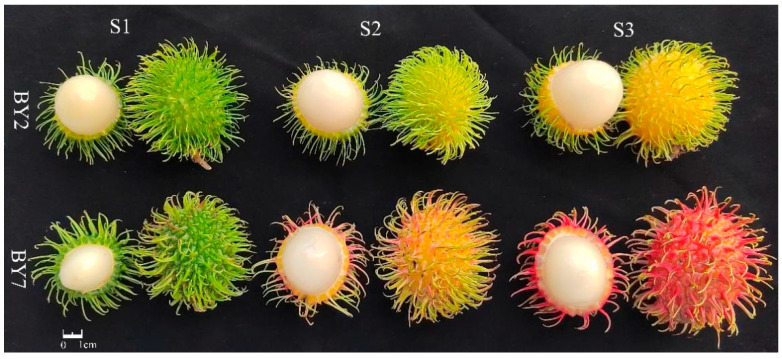
Three growth stages of BY2 and BY7 rambutans.

As shown in Figure 6, unripe (S1, 60 ± 2 days after flowering, 29 ± 5 mm transverse diameter), half-ripe (S2, 90 ± 2 days after flowering, 33 ± 3 mm transverse diameter), and full-ripe fruit (S3, 120 ± 2 days after flowering, 35 ± 3 mm transverse diameter) of BY2 and BY7 rambutans were collected from three random six-year-old trees at the Baoting Tropical Crops Institute (18.6095 N, 109.74039 E). Each growth stage of rambutan had three samples, with at least 45 fruits. Rambutan pulps were separated from seeds, peeled, and juiced by a mix mill (LZ25Easy121, Midea Group Co. Ltd., Guangdong, China) for two min for basic qualities analysis.

### 3.3. Basic Quality Analysis of Rambutans

The soluble sugar content was determined by a UV-Vis spectrophotometer (752 N Plus, Inesa Analytical Instrument Co., Ltd., Shanghai, China) at a wavelength of 620 nm. The titratable acid content was measured by 0.02 mol/L NaOH. A pH meter (PH838, Smart Sensor, Dongguan, China) and a Brix refractometer (FNV-55, Henan Suijing Environmental Protection Technology Co., Ltd., Luoyang, China) were used to determine pH and total soluble solids, respectively. L*, a*, b* values were measured by a spectrophotometer (NS800, Shenzhen Threenh Technology Co., Ltd., Shenzhen, China). The color contribution index (CCI) was calculated as 1000 × a*/(L* × b*).

### 3.4. Sugar and Organic Acid Analysis

The contents of fructose, glucose, and sucrose were determined by HPLC according to GB5009.8-2016 (National Food Safety Standards: Determination of Fructose, Glucose, Sucrose, Maltose and Lactose in Foods, CN) [30]. The contents of malic, lactic, tartaric, citric, succinic, adipic, and fumaric acids were determined by HPLC according to GB5009.157-2016 (National Food Safety Standards: Determination of Organic Acids in Foods, CN) [31].

### 3.5. Amino Acid Analysis

Fifty milliliter pure water was added to a tube with a 2 g sample and two ceramic homogenizers. After 10 min of vortexing and 10 min of ultrasonic extraction, the solution was treated with a filter membrane (0.22 μm) before analysis. The contents of 21 amino acids were determined by UPLC-MS/MS according to GB/T30987-2020 (Determination of Free Amino Acids in Plant, CN) [32].

### 3.6. Widely Targeted Metabolomics Analysis

A UPLC–MS/MS analysis was performed according to Deng et al. [33] and Yin et al. [34] with some modifications. Briefly, UPLC (Nexera X2, Shimadzu, Kyoto, Japan) was equipped with a column (SB-C18, 1.8 μm, 2.1 mm × 100 mm, Agilent) and coupled to a 6500 quadrupole-linear ion trap (QTRAP) mass spectrometer. First, a 2.0 μL filtered sample was loaded and maintained on a column at 40 °C. The flow rate of the mobile phase was 0.35 mL/min, which consisted of solvent A (ultrapure water with 0.1% formic acid) and solvent B (acetonitrile with 0.1% formic acid). The mobile phase in the column was programmed as follows: 0~9 min, 5%~95% B; 9~10 min, 95% B; 10~11.1 min, 95% B~5% B; 11.1~14 min, 5% B. A specific set of multiple reaction monitoring (MRM) transitions were monitored for each period according to the metabolites eluted within this period.

### 3.7. Identification and Quantification of Differential Metabolites

Five crucial MS parameters, including DP (declustering potential), CE (collision energy), RT (retention time), Q1 (precursor ion), and Q3 (product ion), were used to identify the metabolite from the Metware database (Wuhan Metware Biotechnology Co., Ltd., Wuhan, China). The peak area of each metabolite was used for relative quantification analysis. Differential metabolites (DMs) between growth stages of rambutan were selected by fold change (FC) ≥ 2 and |Log_2_FC| ≥ 1.0. A variable importance in projection value (VIP ≥ 1) from the orthogonal projections to latent structures-discriminatory analysis (OPLS-DA) model was then used to screen these metabolites again.

### 3.8. Verification of Potential Taste Biomarkers

The absolute contents of four taste biomarkers, including GABA, Met, Ala, and SMC were determined by UPLC-MS/MS according to GB/T30987-2020 (Determination of Free Amino Acids in Plant, CN) [32]. All analyses were repeated three times.

### 3.9. Statistical Analysis

A principal component analysis (PCA) and hierarchical cluster analysis (HCA) was carried out by R (www.r-project.org (accessed on 12 August 2022)). OriginLab (2019, OriginLab Inc., Northampton, MA, USA) software, which was used for data statistical analysis and graphing.

## 4. Conclusions

In this study, three growth stages of BY2 and BY7 rambutans were compared and profiled, followed by the identification and verification of potential taste biomarkers. We proved that the sugar-acid ratios of the two rambutans were greatly improved between the half-ripe (S2) and full-ripe (S3) stages. However, the taste enhancement of BY2 rambutan mainly relied on the reduction of lactic, citric, and malic acids, while the increase in sucrose and fructose in BY7 rambutan primarily determined the sweet taste improvement at the same growth stages. In addition, the total amino acid contents of the two cultivars increased significantly to reach the maxima of 1438.7 and 1207.2 mg/kg at the S2 stage, respectively. A further UPLC–MS/MS-based widely targeted metabolomics analysis showed that there were 232, 205, 204, and 12 differential metabolites (DMs) in BY2-S1 vs. BY2-S2, BY2-S2 vs. BY2-S3, BY7-S1 vs. BY7-S2, and BY7-S2 vs. BY7-S3, respectively. A correlation analysis between all taste indicators and DMs showed that gamma-aminobutyric acid (GABA) and methionine (Met) could be the sugar-acid ratio and total amino acid biomarkers of BY2 rambutan, respectively. Alanine (Ala) and S-methyl-L-cysteine (SMC) showed positive correlations (Pearson’s r = 0.9999, *p* < 0.01) with total amino acids, and could be potential biomarkers of BY7 rambutan. In addition, UPLC-MS/MS-based quantitative verification exhibited the same variations between biomarkers and related taste qualities during maturation. Our findings not only systematically profiled two rambutan but also provided valuable information for breeding high-taste fruit.

## Data Availability

All data presented in the present research are available on request from the corresponding author.

## Supplementary Materials

The following supporting information can be downloaded at: https://www.mdpi.com/article/10.3390/molecules28031390/s1. Table S1: Basic qualities of BY2 and BY7 rambutans at three growth stages; Table S2: Sugars and organic acids of BY2 and BY7 rambutans at three growth stages; Table S3: Amino acids of BY2 and BY7 rambutan at three growth stages (mg/kg).

## Sample Availability

Samples of the compounds are available from the authors.
