# Supplementary material for: Metabolic Profiling and Potential Taste Biomarkers of Two Rambutans during Maturation
Source: Molecules. 2023 Feb 1;28(3):1390. doi: 10.3390/molecules28031390 (PMC9920857; doi:10.3390/molecules28031390)
Supplement: Supplementary file 1 [file molecules-28-01390-s001.zip › Table S1. Basic qualities of BY2 and BY7 rambutans at three growth stages.pdf]

Table S1. Basic qualities of BY2 and BY7 rambutans at three growth stages

**Cultivar**	**Growth stage**	**Total soluble solid (%)**	**pH**	**Soluble sugar (%)**	**Titratable acid (%)**	**Sugar-acid ratio**	**Color contribution index**
BY2	S1	16.1±0.3[c]	3.8±0.06[c]	12.3±0.9[b]	0.57±0.08[a]	21.7±1.4[b]	-7.2±0.5[a]
	S2	17.4±0.4[b]	4.1±0.07[b]	14.8±0.9[a]	0.52±0.06[a]	28.6±2.8[b]	-1.7±0.3[b]
	S3	17.9±0.3[a]	4.6±0.11[a]	16.4±0.6[a]	0.17±0.01[b]	94.5±9.2[a]	0.3±0.03[c]
BY7	S1	14.2±0.4[c]	3.4±0.03[b]	2.1±0.1[c]	1.0±0.01[a]	2.1±0.1[c]	-14.0±1.1[b]
	S2	17.3±0.2[b]	4.2±0.08[a]	6.4±0.3[b]	0.4±0.02[b]	15.5±2.5[b]	8.4±2.0[a]
	S3	19.9±0.5[a]	4.3±0.04[a]	24.0±1.5[a]	0.3±0.02[c]	74.0±8.2[a]	30.2±5.7[a]

Note: Sugar-acid ratio is soluble sugar divided by titratable acid. Different letters on the number meant significant differences between growth stages in same cultivar ($p<0.05$).
