# Supplementary material for: Metabolic Profiling and Potential Taste Biomarkers of Two Rambutans during Maturation
Source: Molecules. 2023 Feb 1;28(3):1390. doi: 10.3390/molecules28031390 (PMC9920857; doi:10.3390/molecules28031390)
Supplement: Supplementary file 1 [file molecules-28-01390-s001.zip › Table S2. Sugars and organic acids of BY2 and BY7 rambutans at three growth stages.pdf]

Table S2. Sugars and organic acids of BY2 and BY7 rambutans at three growth stages

Cultivar	Growth stage	Sucrose (g/kg)	Fructose (g/kg)	Glucose (g/kg)	Lactic acid(g/kg)	Citric acid(g/kg)	Malic acid(g/kg)	Tartaric acid(mg/kg)	Adipic acid(mg/kg)	Succinic acid(mg/kg)	Fumaric acid(mg/kg)
BY2	S1	70.8 ± 2.7^{b}	19.6 ± 1.8^{a}	18.4 ± 1.0^{a}	12.5 ± 0.6^{b}	5.3 ± 0.3^{a}	6.9 ± 0.9^{ab}	384.0 ± 15.7^{c}	220.4 ± 5.6^{a}	189.9 ± 21.7^{b}	0.20 ± 0.07^{b}
	S2	78.5 ± 2.4^{a}	15.4 ± 0.3^{a}	15.3 ± 0.4^{b}	14.2 ± 0.3^{a}	5.9 ± 0.5^{a}	7.4 ± 0.4^{a}	613.8 ± 12.3^{a}	138.9 ± 20.6^{b}	1028.7 ± 54.6^{a}	0.35 ± 0.04^{a}
	S3	83.9 ± 4.3^{a}	19.6 ± 4.8^{a}	18.5 ± 2.2^{a}	10.2 ± 0.8^{c}	2.0 ± 0.1^{b}	5.7 ± 0.4^{b}	509.4 ± 27.9^{b}	161.6 ± 7.0^{b}	248.4 ± 29.4^{b}	0.35 ± 0.05^{a}
BY7	S1	61.9 ± 0.4^{c}	14.4 ± 0.1^{b}	14.0 ± 1.7^{a}	12.5 ± 1.2^{a}	8.7 ± 0.4^{a}	7.8 ± 0.3^{a}	522.3 ± 16.4^{b}	201.7 ± 4.5^{b}	127.1 ± 15.2^{b}	0.12 ± 0.01^{b}
	S2	82.4 ± 1.7^{b}	16.0 ± 1.3^{b}	15.8 ± 1.4^{a}	8.3 ± 0.4^{b}	4.7 ± 0.1^{b}	5.6 ± 0.2^{c}	566.0 ± 15.5^{a}	202.3 ± 6.1^{b}	206.7 ± 18.7^{a}	0.23 ± 0.04^{a}
	S3	97.2 ± 2.9^{a}	18.5 ± 0.6^{a}	16.7 ± 1.7^{a}	9.3 ± 0.8^{b}	3.5 ± 0.1^{c}	6.3 ± 0.1^{b}	493.2 ± 13.2^{b}	213.7 ± 1.9^{a}	261.3 ± 19.1^{a}	0.22 ± 0.04^{a}

Note: Different letters on the number meant significant differences between growth stages in same cultivar (p<0.05).
