# Supplementary material for: Metabolic Profiling and Potential Taste Biomarkers of Two Rambutans during Maturation
Source: Molecules. 2023 Feb 1;28(3):1390. doi: 10.3390/molecules28031390 (PMC9920857; doi:10.3390/molecules28031390)
Supplement: Supplementary file 1 [file molecules-28-01390-s001.zip › Table S3. Amino acids of BY2 and BY7 rambutan at three growth stages.pdf]

Table S3. Amino acids of BY2 and BY7 rambutan at three growth stages (mg/kg)

Cultivar	Growth Stage	Ala	Arg	Asn	Asp	GABA	Gln	Glu	Gly	His	Ile	Leu	Lys	Met	Phe	Pro	Ser	Thr	Tyr	Val	Total amino acids
BY2	S1	73.4±6.1^{c}	18.6±1.7^{c}	18.6±1.3^{c}	69.8±1.7^{a}	62.2±4.5^{b}	105.7±17.8^{b}	49.5±6.5^{b}	0.2±0.2^{c}	3.9±0.3^{c}	9.3±1.0^{b}	8.4±0.9^{b}	102.6±17.0^{b}	11.1±1.5^{c}	5.0±0.7^{b}	4.9±0.3^{c}	25.8±2.0^{c}	13c6±0.3^{c}	14.7±1.2^{c}	15.9±1.2^{c}	613.3±45.2^{b}
	S2	368.9±26.1^{b}	40.9±4.1^{a}	61.6±7.5^{a}	45.5±7.2^{b}	75.2±6.6^{b}	229.8±23.0^{a}	65.4±5.7^{a}	1.4±0.7^{b}	20.3±2.8^{a}	10.5±0.7^{a}	10.0±1.0^{a}	221.5±21.9^{a}	32.3±1.4^{a}	15.1±4.2^{a}	12.7±3.1^{a}	70.6±9.8^{a}	38.4±3.5^{a}	68.0±4.9^{a}	50.6±6.3^{a}	1438.7±110.7^{a}
	S3	467.8±31.4^{a}	30.2±1.0^{b}	44.1±2.5^{b}	35.4±6.2^{c}	198.3±11.1^{a}	111.7±5.3^{b}	7.1±1.5^{c}	2.4±0.7^{a}	9.3±0.4^{b}	9.0±0.7^{b}	8.4±0.7^{b}	106.8±7.0^{b}	17.2±1.0^{b}	4.1±0.7^{b}	9.4±0.1^{b}	52.8±1.9^{b}	32.3±1.1^{b}	20.5±0.6^{b}	25.1±0.6^{b}	1192.0±105.8^{a}
BY7	S1	52.5±12.5^{b}	28.4±3.4^{a}	17.6±1.7^{c}	55.0±3.9^{a}	48.3±8.5^{c}	135.4±16.6^{a}	42.7±5.9^{a}	0±0^{b}	4.0±0.4^{b}	6.6±0.6^{b}	6.0±0.3^{b}	129.9±14.9^{a}	9.6±0.8^{c}	3.6±0.4^{c}	3.6±0.2^{c}	26.3±1.3^{b}	11.6±0.5^{c}	13.2±1.3^{c}	12.9±1.1^{c}	607.4±50.4^{b}
	S2	448.6±25.1^{a}	28.7±2.3^{a}	39.4±1.9^{a}	41.3±6.8^{b}	136.7±18.1^{b}	128.2±6.3^{a}	43.3±7.0^{a}	2.0±0.4^{a}	9.7±0.6^{a}	10.0±1.2^{a}	9.2±1.1^{a}	123.4±6.4^{a}	17.1±1.9^{a}	6.8±0.7^{a}	11.9±1.0^{a}	62.1±1.8^{a}	31.1±0.8^{a}	26.4±2.7^{a}	31.4±1.9^{a}	1207.2±97.9^{a}
	S3	436.9±32.4^{a}	29.3±6.2^{a}	35.5±2.1^{b}	35.5±5.6^{b}	174.6±14.5^{a}	124.2±15.4^{a}	32.6±2.0^{a}	2.2±0.2^{a}	9.1±1.6^{a}	9.4±0.4^{a}	8.5±0.5^{a}	122.3±12.8^{a}	11.7±1.2^{b}	4.8±0.1^{b}	9.7±0.9^{b}	61.4±5.0^{a}	28.2±2.2^{b}	22.8±2.9^{b}	25.4±1.3^{b}	1184.0±107.4^{a}

Note: Different letters on the number meant significant differences between growth stages in same cultivar (p<0.05).
